# Effect of thymoquinone on transient receptor potential melastatin (TRPM) channels in rats with liver ischemia reperfusion model in rats

**DOI:** 10.22038/IJBMS.2023.71990.15647

**Published:** 2024

**Authors:** Kerem Caglar, Recep Dokuyucu, Gokhan Agturk, Cemil Tumer, Okan Tutuk, Hatice Dogan Gocmen, Hasan Gokce, Zeynel Abidin Tas, Oguzhan Ozcan, Bulent Gogebakan

**Affiliations:** 1 Department of Physiology, School of Medicine, Hatay Mustafa Kemal University, Hatay, Turkey; 2 Department of Physiology, School of Medicine, Atlas University, Istanbul, Turkey; 3 Department of Physiology, Institute of Health Sciences, Istanbul University Cerrahpasa, Istanbul, Turkey; 4 Department of Physiology, School of Medicine, Halic University, Istanbul, Turkey; 5 Department of Pathology, School of Medicine, Inonu University, Malatya, Turkey; 6 Department of Biochemistry, School of Medicine, Hatay Mustafa Kemal University, Hatay, Turkey; 7 Department of Medical Biology, Private Sevgi Hospital, Osmaniye, Turkey

**Keywords:** Cation channels, Injury, Ischemia-reperfusion, Thymoquinone, TRPM

## Abstract

**Objective(s):**

We aimed to investigate the levels of transient receptor potential melastatin (TRPM) gene expression, and the antioxidant and histopathologic effect of thymoquinone (Tmq) in the hepatic I/R rat model.

**Materials and Methods:**

Fifty Wistar rats were divided into 5 groups. Group 1: Control; Group 2: Sham; Group 3: Hepatic I/R (45 min/45 min); Group 4: Tmq (50 mg/kg); Group 5: Tmq+I/R (ten days before I/R at the dose of 50 mg/kg of Tmq). The hepatic I/R (45min/45min) model was performed at the portal vein and the hepatic artery with atraumatic vascular clamp in the ischemia groups. The liver tissues and blood samples that were taken at the end of the study were evaluated for histopathologic and biochemical analysis. Besides *TRPM* gene expression levels were determined in liver tissues. It was seen that cellular swelling, congestion, PNL, and apoptosis parameters statistically decreased in Tmq and Tmq+I/R groups in comparison with the I/R group in histopathological evaluation.

**Results:**

It was observed that biochemical parameters, AST, ALT, GGT, LDH, creatinine, and urea levels significantly increased in the I/R group as compared with, sham, Tmq, and Tmq+I/R groups. It was found that *TRPM2,6,7,8* gene expression decreased significantly in Tmq+I/R groups as compared to the I/R group.

**Conclusion:**

We showed that thymoquinone can inhibit the entry of Ca+2 into the cell by decreasing *TRPM2,6,7,8* gene expression. Based on our findings, we think that Tmq application in the treatment of liver diseases due to I/R damage may be important in terms of both ischemia and apoptosis and can also be used in the treatment of liver-related diseases.

## Introduction

Prolonged ischemia due to ischemia-reperfusion (I/R) injury, and lack of arterial or venous blood occurs when circulation is restored in organs and tissues. This greatly affects oxygen-sensitive aerobic cells, which use mitochondrial oxidative phosphorylation as an energy source. For this reason, I/R damage is more likely to occur in organs and tissues that use aerobic metabolism (1). 

I/R injury is a common condition encountered during or after hypovolemic shock, chronic liver diseases, major tumor resections, surgical intervention to hepatic trauma, vascular reconstructions, and hepatic transplantation. Ischemia is the most common type of cell injury in clinical medicine. Therefore, it causes life-threatening problems in many tissues and organs, especially the brain, heart, kidneys, and liver. Particularly in hepatic ischemia models, many studies have been carried out and many agents have been used to minimize the resulting ischemic liver injury and subsequent reperfusion injury. However, since liver I/R injury has not been clarified yet, it is important to understand the pathophysiology of liver I/R-related diseases in the clinic and to reveal different treatment modalities for these diseases (1, 2).

Thymoquinone (Tmq) is a monoterpene and the major compound of *Nigella sativa* seeds, a promising medicinal plant with many therapeutic effects. As a result of the investigations, 38.20% fat, 31.94% carbohydrates, 20.85% protein, 7.94% fiber, and 4.64% water are the content of *N. sativa* seeds (3, 4). Tmq has also shown antioxidant effects. It inhibited inflammation in animal models and cell culture systems (5, 6). Despite studies showing that Tmq is anticancer, antioxidant, antibacterial, antifungal, antiparasitic, and antiasthmatic, its mechanism of action has not yet been fully elucidated (3, 7, 8). 

Studies have increased the possibility of Transient Receptor Potential (TRP) proteins being a new Ca^+2 ^permeable cation channel. TRP channels either act directly as Ca^+2^ entry channels in plasma membranes or assist in the change in cytosolic free Ca^+2 ^channels that change the membrane potential, which is the driving force for the modulation of Ca^+2 ^entry channels (9). TRP channels play a role in many important cellular processes such as Ca^+2^-Mg^+2^ transmission, regulation of blood pressure, perception of taste, smell, sound, gene expression and secretion, apoptosis, and in many important mechanisms such as the commonly known secondary messenger mechanism and ion entry and exit (10). 

As a result of genetic studies, TRP channels were divided into 7 subfamilies according to their amino acid similarities. These are TRPC (canonical), TRPM (melastatin), TRPV (vanilloid), TRPA (anycrine-rich protein), TRPP (polycystin), TRPML (mucolipin), and TRPN (nomic). The activation mechanisms of these channels and the organs in which they are located are different. For example, melastatin is mostly TRP, found in the brain and neuron cells (11). A total of 8 sub-members of the TRP Melastatin (TRPM) family, defined as TRPM1-TRPM8, have been reported. Sub-members of TRPM are classified under 4 groups according to their sequence similarities: TRPM1,3, TRPM2,8, TRPM4,5, and TRPM6,7. TRPM4,5 channels are not permeable to Ca^+2^, while TRPM6 and 7 show high permeability to Ca^+2^ and magnesium. Unlike TRPC and TRPV channels, TRPM channels do not contain ankyrin repeats. No activator has yet been demonstrated for the TRPM1 channel. It has been suggested that the TRPM1 channel is a constitutively active Ca^+2^ entry channel (11, 12). TRPM3 expression has been demonstrated in the kidney, brain, testis, and spinal cord (11, 12).

In our study, we planned to investigate the effects of thymoquinone on ischemia/reperfusion and *TRPM 2,6,7,8* ion channels in liver tissues by creating an I/R model in the liver. Considering the important role of TRPM channels in ischemia and apoptosis, the clarification of this issue and therefore the relationship between the expression of *TRPM 2,6,7,8* channels and thymoquinone will allow evaluating its potential in the diagnosis and treatment of both ischemia and apoptosis and liver-related diseases. Considering the important role of TRPM channels in ischemia and apoptosis in the presented study; it is aimed to contribute to the evaluation of both the relationship of TRPM channel expressions with Tmq and the potential of Tmq in the diagnosis and treatment of ischemia, apoptosis, and liver-related diseases.

## Materials and Methods

This experimental study was carried out in the laboratory of Mustafa Kemal University, Faculty of Medicine Experimental Animals Research Center (protocol number 2014-5/9, Mustafa Kemal University Animal Experiments Ethics Committee). The histopathological and biochemical analysis of our study was carried out in the laboratories of Mustafa Kemal University Research and Application Hospital, Department of Pathology, and Department of Biochemistry. Molecular and genetic analyses of our study were performed in the laboratory of Mustafa Kemal University Faculty of Medicine, Department of Medical Biology and Molecular Genetics. The study was planned by forming 5 groups of 50 adult male Wistar Albino rats weighing 300-400 g (Table 1). During the experiment, rats were kept in wire cages in rooms with regular light-dark treatment (12:12), 20-24 ^°^C temperature, and standard humidity. Standard pellet feed and tap water were used to feed the animals.

***Experimental study model***

Anesthesia of the subjects was provided by giving 80 mg/kg ketamine and 12 mg/kg xylazine, and maintenance doses were administered when necessary. Fifteen minutes after drug injection, the hairs on the anterior abdominal wall were shaved and the abdominal skin was wiped with povidone-iodine, and skin antisepsis was applied.

A laparotomy was performed with an incision of approximately 3-4 cm from the midline. After the organs in the abdomen became visible, the blood flow to the left and middle lobes of the liver was cut off by exploring the portal vein and hepatic artery in groups other than the control group and by an atraumatic vascular clamp. Thus, segmental (70%) and non-lethal hepatic ischemia were created. Ischemia was applied for 45 min with the help of an atraumatic vascular clamp. A color change on the liver surface was observed with ischemia.

To prevent thrombosis that may occur in the vein due to tightening of the suture, heparin at a dose of 600 IU/kg was administered intravenously before the initiation of ischemia. Meanwhile, the exposed abdomen was covered with a sponge soaked in warm saline, clamps were removed at the end of 45 min, and the ischemia was terminated. The laparotomy incision of the rats, which started the reperfusion phase (45 min), was closed with 3/0 atraumatic silk suture, 5 ml blood samples were taken intracardiac at the end of 45 min, and then the subjects were euthanized by cervical dislocation (14).

As a result of the study, some of the liver tissues were taken into cryo tubes to be stored at -80 ^°^C for gene expression studies. Then, tissue samples were taken from the left lobe of the liver and taken into 10% formol solution to be sent for histopathological examination. Some of them were placed in cryo tubes to be stored at -80 ^°^C for gene expression studies. Samples were blocked according to the histological methods. Sections were cut from paraffin blocks and stained with Hematoxylin-Eosin (H&E). The prepared preparations were examined under a research microscope (Olympus CX31-Japan) and their photographs were taken at 40 X magnification. Then, the severity of the damage was evaluated according to the Suzuki Score (15).

Aspartate aminotransferase (AST) and alanine aminotransferase (ALT) lactate dehydrogenase (LDH) levels were determined in the micro-ELISA device in order to detect liver damage. Tumor necrotizing factor alpha (TNF-a) and interleukin 6 (IL-6) levels in plasma were determined by mouse TNF-a ELISA kit and mouse IL-6 ELISA kit purchased from BD BioSciences. Thiol level was measured with the spectrophotometric method defined by Erel and Neselioglu (16). Total antioxidant status (TAS) and total oxidant status (TOS) levels in serum were measured spectrophotometrically according to Erel’s method (17, 18). Additionally, the OSI value of each participant was calculated by proportioning TOS to TAS (19).

Molecular analysis evaluations were performed to demonstrate the expression of TRPM channels. mRNA expression in tissues, RNA isolation, cDNA extraction, and Quantitative real-time Reverse Transcriptase PCR were examined. The results of PCR were compared with each other and it was decided which gene expression increased or decreased.

***Quantitative real-time PCR (qRT-PCR)***

TRPM2,6,7,8 and ‘housekeeping’, b-Actin (Ella Biotech, Deutschland) gene transcription levels were determined by quantitative RT-PCR method (QIAGEN Rotor-Gene Q, Germany)(Table 2). Quantitative values were determined according to the normalization coefficient. 

***Statistical analysis***

Statistical evaluations of biochemical and histopathological results were performed using v. 5.0 statistical package program. Data are presented as Mean±standard error. In statistical analysis, One-way ANOVA test (*post hoc* Bonferroni and Student’s t-test) was used to compare groups with regular distribution according to Kolmogorov-Smirnov distribution analysis, and Kruskal-Wallis test (*post hoc* Dunn’s test) was used to compare groups with uneven distribution. *P*<0.05 values were considered statistically significant.

## Results

A comparison of liver function test parameters between groups is shown in Table 3. A statistically significant increase was found in the I/R group when compared to the control, Tmq, and Tmq+I/R groups in the levels of biochemical parameters AST, ALT, GGT, and LDH (*P*<0.05). A statistically significant increase was found in the I/R group when compared to the control, Tmq, and Tmq+I/R groups in the levels of biochemical parameters AST, ALT, GGT, and LDH (*P*<0.05). When compared to the control group, there was a significant increase in AST, ALT, GGT, and LDH levels in the I/R group (*P*<0.001). There was a significant increase in ALT level in the Tmq group when compared with I/R and Tmq+I/R groups (*P*<0.001). There was a significant increase in GGT level in the Tmq group when compared with I/R and Control groups (*P*<0.01). When compared with I/R and Control groups, there was a significant decrease in AST and LDH levels in the Tmq group (*P*<0.001). When compared with the I/R group, there was a significant decrease in AST and ALT levels in the Tmq+I/R group (*P*<0.01). When compared with the I/R group, there was a significant increase in GGT and LDH levels in the Tmq+I/R group (*P*<0.001). When compared with the Tmq+I/R group, there was a significant decrease in AST and ALT levels in the control group (*P*<0.001). When compared with the Tmq+I/R group, there was a significant decrease in GGT and LDH levels in the control group (*P*<0.01)(Table 3).

***Findings related to plasma TNF-a and IL-6 levels***

In our study, the plasma levels of proinflammatory cytokines TNFa and IL-6 were determined in the groups, and the effect of Tmq administration on these cytokine levels and therefore on early reperfusion injury was investigated. Plasma TNF-a and IL-6 levels of the I/R group were found to be statistically significantly higher than the levels of the control and sham groups (*P*<0.001); In the Tmq+I/R group, in which Tmq was applied 10 days before the I/R protocol, this level decreased compared to the values of the I/R group (*P*<0.001). It was observed that plasma cytokine levels in the Tmq group were applied 10 days before the I/R protocol approached the levels in the control and sham groups (Table 4).

***Kidney function tests***

A comparison of renal function test parameters between groups is shown in Table 5. A statistically significant increase was found in the I/R group when compared with the control, Tmq, and Tmq+I/R groups in the creatine and urea levels of biochemical parameters (*P*<0.05). When compared to the control group, there was a significant increase in creatinine and urea levels in the I/R group (*P*<0.01). A significant decrease in creatinine and urea levels was found in the Tmq group when compared with the I/R group (*P*<0.01). When compared with the I/R group, there was a significant decrease in creatinine levels in the Tmq+I/R group (*P*<0.05). When compared with the Tmq+I/R group, there was a significant decrease in creatinine levels in the Tmq group (*P*<0.01). When compared with the Tmq+I/R group, there was a significant decrease in creatinine levels in the control group (*P*<0.01)(Table 5).

***Evaluation of oxidative stress parameters***

Comparison of oxidative stress parameters between groups is shown in Table 6. The I/R group showed a statistically significant increase in oxidative stress parameters compared to the control group. However, Tmq and Tmq+I/R groups showed a statistically significant decrease in oxidative stress parameters compared to the I/R group (*P*<0.001)(Table 6).

***Histopathological results***

**Figure 1:**
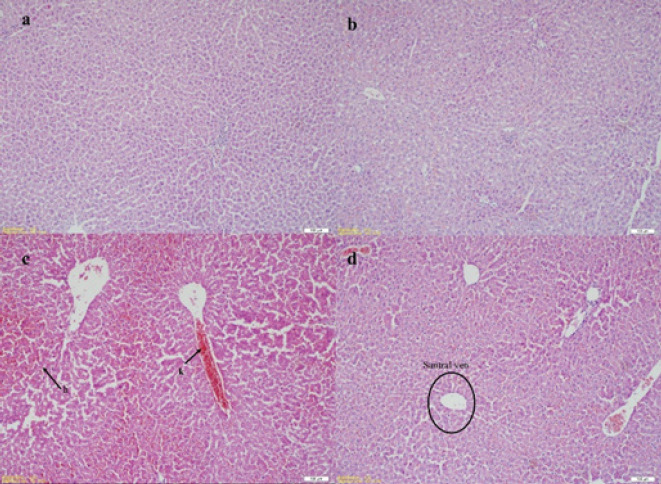
Histopathological appearance of rat livers

Various cellular changes were observed between the groups by examining the sections of liver tissue stained with H&E staining. In the histopathological evaluation, cellular swelling, congestion, polymorphonuclear leukocytes (PMN), and apoptosis values were significantly increased in the I/R group compared to the control and sham groups (*P*<0.01). Statistically significant decreases were observed in cellular swelling, congestion, PMN, and apoptosis values in the Tmq and Tmq+I/R groups when compared to the I/R group (*P*<0.01)(Table 7, Figure 1).

***Gene results***

**Figure 2:**
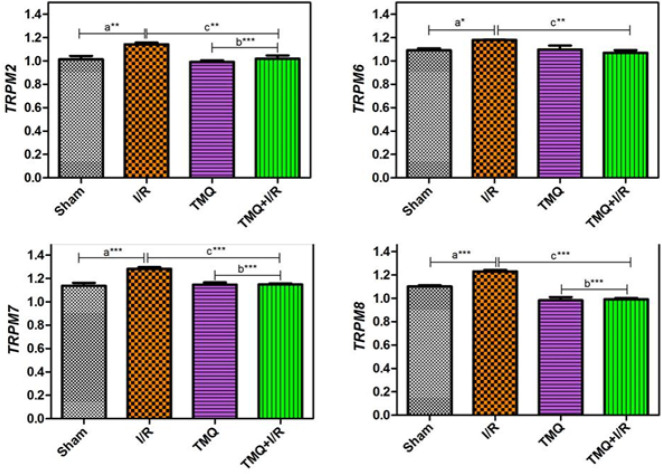
Comparison of gene results of the study groups

As a result of real-time PCR, the ratios of genes present on the agarose gel were examined to evaluate which genes increased or decreased. Numerical data indicating decreases, normal levels, and increases were extracted. TRPM2, 6, 7, and 8 gene expression levels are shown in the table, and the expression levels of each gene in the individual groups are shown graphically (Figure 2). TRPM2, 6, 7, and 8 expressions were found to be statistically significantly decreased in the Tmq+I/R group when compared to the I/R group (*P*<0.01, *P*<0.001, respectively)(Table 8).

**Table 1:** Experimental groups and application doses used in the study (13)

**Groups**	**Number of Subjects**
Control Group	10
Sham	10
Liver Ischemia/Reperfusion group (45 min I / 45 min R)	10
Tmq group (50 mg/kg Tmq by oral gavage 10 days before) (13) (purity ≥ 98%; Sigma-Aldrich, St. Louis, MO, USA)	10
Tmq + I/R group (50 mg/kg Tmq + 45 min I / 45 min R by oral gavage for 10 days before I/R).	10
**Total**	50

**Table 2:** Primer sequences used in quantitative real-time PCR (qRT-PCR) analysis of the study groups

b-Actin	Left	5’-CCC GCG AGT ACA ACC TTC T-3
b-Actin	Right	5’-CGT CAT CCA TGG CGA ACT-3
TRPM2	Left	5’-AAT TTG CTC ATC GCC ATG TT-3
TRPM2	Right	5’-GAT CTG GTC TGT GTG CTC CTG-3
TRPM6	Left	5’-GCA AGA ACT GGC TTT CCG TG-3
TRPM6	Right	5’-ATC CGG GTC CTC TTG CAT CT-3
TRPM7	Left	5’-AGA CGC TTT CCG ATA GAT GG-3
TRPM7	Right	5’-CTA TCC AGG ATT TCT GGG ACA T-3
TRPM8	Left	5’-GCC CAG TGA TGT GGA CAG TA-3
TRPM8	Right	5’-GGA CTC ATT TCC CGA GAA GG-3

**Table 3:** Comparison of liver function test parameters of the study groups (Mean±SD)

	**Control**	**Sham**	**Tmq**	**I/R**	**Tmq + I/R**
AST (U/l)	145.8±45.1^c***^	155.4±39.3	137.9±24.7 ^b,c***^	2155.0±236.5 ^a***^	1389.0±171.9 ^b**^
ALT (U/l)	51.80±9.7 ^c***^	67.02±9.1	54.1±4.9 ^b,c***^	1076.0±72.3 ^a***^	819.9±61.7 ^b**^
GGT (U/l)	1.83±0.30 ^c**^	1.85±0.26	2.28±0.28 ^b,c**^	9.12±0.85 ^a***^	4.87±0.51 ^b***^
LDH (mg/dl)	697.8±211.3 ^c**^	872.0±272.3	442.7±90.8 ^b,c***^	4595.0±424.1 ^a***^	2566±376.1 ^b***^

I/R: Ischemia/Reperfusion; Tmq: Thymoquinone. AST: Aspartate Aminotransferase; ALT: Alanine Aminotransferase; GGT: Gamma Glutamyl Transferase; LDH: Lactate Dehydrogenase, * *P<*0.05; ** *P<*0.01; *** *P<*0.001; ^a^ vs Control; ^b^ vs I/R; ^°^ vs Tmq+I/R

**Table 4:** Comparison of the parameters of plasma TNF-and IL-6 levels of the study groups (Mean±SD)

	**Control**	**Sham**	**Tmq**	**I/R**	**Tmq + I/R**
TNF-α (pg/ml)	57.14±8.680 ^c**^	50.23±11.51	53.49±8.288 ^b,c**^	161.8±5.107 ^a***^	93.63±6.340 ^b***^
IL-6 (pg/ml)	46.75±6.994 ^c**^	50.63±7.481	41.50±6.156 ^b,c***^	110.6±12.81 ^a***^	74.63±5.010 ^b***^

I/R: Ischemia/Reperfusion; Tmq: Thymoquinone. TNF-α: Tumor Necrotizing Factor Alpha; IL-6: Interleukin-6; ** *P<*0.01; *** *P<*0.001; ^a^ vs Control; ^b^ vs I/R; ^°^ vs Tmq+I/R

**Table 5:** Comparison of renal function test parameters of the study groups (Mean±SD)

	**Control**	**Sham**	**Tmq**	**I/R**	**Tmq + I/R**
Creatine (mg/dl)	0.37±0.04 ^c**^	0.40±0.00	0.47±0.01 ^b,c**^	0.75±0.04 ^a***^	0.63±0.03^ b*^
Urea (mg/dl)	17.44±0.2	18.24±0.9	18.30±1.1^ b**^	25.28±1.9^ a***^	22.04±0.6

I/R: Ischemia/Reperfusion; Tmq: Thymoquinone. ^a^ vs Control; ^b^ vs I/R; ^°^ vs Tmq+I/R. ** *P<*0.01; *** *P<*0.001

**Table 6:** Comparison of oxidative stress parameters of the study groups (Mean±SD)

	**Control**	**Sham**	**Tmq**	**I/R**	**Tmq + I/R**
TAS (mmol/l)	0.87±0.04	0.90±0.05	1.40±0.13 ^a,b**^	0.75±0.07	1.22±0.07 ^b**^
TOS (umol/l)	19.07±6.04	21.56±4.41	14.75±1.85^b,c**^	48.99±4.44^ a***^	34.19±5.07
OSI (TOS/TAS)	22.21±6.56	23.17±3.58	11.91±2.04^ b***^	69.16±9.03^ a***^	27.47±4.01^ b***^

I/R: Ischemia/Reperfusion; Tmq: Thymoquinone. TAS: Total Antioxidant Level; TOS: Total Oxidant Level; OSI: Oxidative Stress Index. ^a^ vs Control; ^b^ vs I/R; ^°^ vs Tmq+I/R. ** *P<*0.01; *** *P<*0.001

**Table 7:** Comparison of histopathological results of the study groups (Mean±SD)

	**Control**	**Sham**	**Tmq**	**I/R**	**Tmq + I/R**
**Cellular swelling**	1.17±0.41 ^c**^	1.57±0.53	1.14±0.38 ^b,c**^	2.88±0.64 ^a**^	1.00±0.53 ^b**^
**Steatosis/lipoid degeneration**	0.00±0.00	0.00±0.00	0.00±0.00	0.00±0.00	0.00±0.00
**Sinusoidal congestion**	2.83±0.75^c**^	2.00±1.00	1.43±0.53^b,c**^	3.75±0.46^a**^	2.38±0.52^b**^
**Hemorrhage**	0.00±0.00	0.00±0.00	0.00±0.00	0.00±0.00	0.13±0.35
**Inflammatory cell infiltration**	0.67±0.52 ^c**^	1.29±0.49	1.43±0.53^b,c**^	1.88±0.64^a**^	1.13±0.35^ b**^
**Lobular necrosis**	0.00±0.00	0.00±0.00	0.00±0.00	0.00±0.00	0.00±0.00
**Apoptosis**	0.17±0.41	0.29±0.49	0.00±0.00	0.50±0.53 ^a**^	0.00±0.00

I/R: Ischemia/Reperfusion; Tmq: Thymoquinone. ** *P<*0.01; ^a^ vs Control; ^b^ vs I/R; ^°^ vs Tmq+I/R

a, b) Normal liver histological appearance in control (a) and sham group (b)(H&E, x100). c, d) Ischemic changes, sinusoidal congestion, cellular swelling, and histopathological appearance of inflammatory cell infiltration in the I/R (c) group and Tmq+I/R (d) group (H&E, x100)

**Table 8:** Comparison of gene expressions of the study groups (Mean±SD)

**Gene**	**Sham**	**I/R**	**Tmq**	**Tmq +I/R**
*TRPM2*	1,014±0,028	1,143±0,013 ^a**^	0,991±0,014 ^b***^	1,020±0,026 ^c**^
*TRPM6*	1,092±0,015	1,180±0,001^ a*^	1,097±0,034	1,071±0,022 ^c**^
*TRPM7*	1,138±0,024	1,282±0,014^ a***^	1,148±0,019^ b***^	1,150±0,008 ^c***^
*TRPM8*	1,102±0,010	1,230±0,012^ a***^	0,982±0,028^ b***^	0,992±0,011 ^c***^

I/R: Ischemia Reperfusion,* *P<*0.05; ** *P<*0.01; *** *P<*0.001 a: Sham vs I/R, b: Tmq vs Tmq+I/R, c: I/R vs Tmq+I/R

## Discussion

Experimental studies have been carried out using many different agents to prevent or minimize ischemic liver injury and subsequent reperfusion injury in hepatic ischemia models. These studies were largely prepared to examine the effects of antioxidant and anti-inflammatory substances. This shows that the damage can be reduced by using antioxidant and anti-inflammatory substances. For this purpose, the protective effects of enzymatic and plant-derived antioxidants against oxidative damage were investigated by experimental studies. A study showed that pentoxifylline and L-arginine may have a protective effect on liver I/R damage (9). Tolba *et al*. investigated the protective effect of vitamin E and caffeic acid phenethyl ester (Cape) on liver I/R damage and determined that they reduced the damage by protecting the integrity of the glucose and pentose phosphate pathway in the tissue, keeping the citric acid cycle active and supporting energy production (20). Research revealed that a dose of 5000 mg. kg of Sumac has a protective effect on the liver when administered intraperitoneally before liver I/R damage (21). Another study found that grape seed extract treatment reduced hepatic I/R injury in rats. It has been determined that grape seed extract reduces HI-induced organ damage by inhibiting neutrophil infiltration and regulating the release of inflammatory mediators with its effect on oxidant-antioxidant balance (22). Researchers examined the effect of papaverine on liver I/R injury in rats, and it was determined that the rats in the study group had significantly reduced I/R injury in all other criteria, except for focal necrosis and hydropic swelling in the parenchyma (23). Abuzinadah and Ahmad showed in their study that thymoquinone has a strong antioxidant effect and this effect is due to its ability to scavenge free radicals (24). Therefore, in our study, we investigated the effectiveness of thymoquinone, which has proven antioxidant activity in the liver I/R injury model. 

Thymoquinone has been used in a wide variety of dose ranges and dose numbers in many experimental studies in rats. Abd El-Ghany *et al*. preferred 45 min of ischemia and 60 min of reperfusion time during the experiment to create hepatic I/R injury. They also determined the antiapoptotic effect of thymoquinone by giving 5, 20, and 50 mg/kg doses to the subjects for 10 days (25). Another study found that thymoquinone had a significant effect on I/R damage by giving 5, 20, 50, and 100 mg/kg doses, while high doses (50 and 100 mg/kg) significantly decreased the GSH content after reperfusion (26). We also showed that oral administration of 50 mg/kg thymoquinone for 10 days before the administration can prevent hepatic I/R damage. We think that this effect is probably due to the oxidative stress-reducing effect of thymoquinone, as well as reducing TNF-a and IL-6 levels, and TRPM gene expression levels in the liver. 

Although the duration of ischemia varies according to personal preference, experimental models have different applications in terms of applied ischemia and reperfusion times. Bayramoglu *et al*. stated that the time required for liver damage is one hour of reperfusion after 45 min of ischemia (27). Researchers showed that 60 min of ischemia and 45 min of reperfusion showed the effect of propofol in the liver ischemia-reperfusion model they created with reperfusion (28). In our study, based on these data, 45 min of ischemia and 45 min of reperfusion were planned upon the determination of hepatic IRH formation biochemically and histopathologically, and gene expression and the experimental model was applied within this framework. Different techniques have been used to evaluate liver functions after I/R injury. Currently, the most commonly used determinations are AST, ALT, and LDH. 

Researchers showed that they created experimental liver IR damage, and claimed that serum AST and ALT values increased and the damage caused by free radicals in the tissue after I/R may cause these values to increase (29). A study found that serum AST and ALT values increased in the IR study on the liver. In addition, necrosis, sinusoidal enlargement, and PMNL infiltration were observed in hepatocytes (30).

Yildirim *et al*. observed an increase in serum AST and ALT values after KC IR in rats (31). In our study, creatinine and urea levels, which are indicators of the effects of AST, ALT, GGT, LDH, and liver IR on the distant organ kidney, which are biochemical parameters that are indicators of liver damage, were examined. When AST, ALT, GGT, LDH, creatinine, and urea levels were compared with the control, pseudo surgery, Tmq, and Tmq+I/R groups, a statistically significant increase was found in the I/R group (*P*<0.05). Plasma TNF-a and IL-6 levels of the I/R group were found to be statistically significantly higher than the levels of the control and sham groups (*P*<0.001); In the Tmq+I/R group, in which Tmq was applied 10 days before the I/R protocol, this level decreased compared to the values of the I/R group (*P*<0.001). It was observed that plasma cytokine levels in the Tmq group were applied 10 days before the I/R protocol approached the levels in the control and sham groups. This suggests that Tmq can significantly reduce liver tissue damage. It has been shown that Ca^+2 ^concentration increases in cells exposed to I/R. The abnormal increase in the intracellular Ca^+2^ concentration causes a cytotoxic effect, the mechanism of which is not fully explained. 

One of the structures that play a role in determining the amount of intracellular Ca^+2^ is TRPM channels. It has been determined that TRPM channels are involved in the functioning of many mechanisms such as apoptosis. It has been understood that this is achieved thanks to the active role of some TRPM channels in cell division (32). There are some hypotheses regarding the increase in Ca^+2^ during ischemia. After ischemic damage, the cell has to divide and the old cell that will go to apoptosis or autophagy will need Ca^+2^ and Mg^+2^ for this division. In addition, the increase in Ca^+2^ triggers the formation of microfibrils. As a result of this situation, Ca^+2^ migrates from the trauma environment; it is necessary for the destruction and regeneration of microfibrils. In this case, the primary molecular change in the cell is the increase in the number of TRPM channels. The main reason for the decrease in the amount of TRPM during reperfusion is to ensure the intake of Ca^+2^ into the cell, which is necessary for the reactions in the cells during ischemia. TRPM expression is decreased due to changes in reperfusion showing the disappearance of ischemia. If a Ca^+2^ channel blocker is given before this picture in tissues that have been damaged by I/R, it will ensure that the negative picture that will be caused by I/R is eliminated (32, 33). In our study, it was observed that thymoquinone, which we administered for treatment, could act through TRPM channels in I/R injury. *TRPM 2,6,7,8* expressions were found to be statistically significantly decreased in the thymoquinone+I/R group compared to the I/R group. 

## Conclusion

Administration of thymoquinone in the treatment of diseases causing I/R in the liver (hypovolemic shock, chronic liver diseases, large tumor resections, surgical intervention in hepatic trauma, vascular reconstructions, and hepatic transplantation, etc.). Our results need to be confirmed by clinical studies in order to be used in the treatment of ischemia and apoptosis as well as liver-related diseases. In addition, in our study, we showed that thymoquinone reduces the *TRPM 2,6,7,8* gene expression level and inhibits the entry of Ca^2+^ into the cell. This situation recalls the idea that thymoquinone has a decreasing effect on cellular damage due to I/R. We think that the damage in the liver ischemia-reperfusion model improved biochemically and histopathologically with thymoquinone treatment, and that the decrease in oxidative stress parameters and TRPM channels may be the mediator of this situation. Our results need to be confirmed by supporting clinical studies.

## Authors’ Contributions

K C and R D conceived and designed the study. G A, HD G, O T, H G, O O, B G, and C T collected the data. GA, HD G, O T, H G, O O, B G, and C T analyzed the data. KC, GA, and R D wrote the manuscript. All authors approved the final manuscript.

## Data Availability

The data that was utilized to corroborate the outcomes of this research have been incorporated within the manuscript and can be freely accessed.

## Conflicts of Interest

None.

## References

[1] Mohamadian M, Parsamanesh N, Chiti H, Sathyapalan T, Sahebkar A (2022). Protective effects of curcumin on ischemia/reperfusion injury. Phytother Res.

[2] Aamani N, Bagheri A, Masoumi Qajari N, Malekzadeh Shafaroudi M, Khonakdar-Tarsi A (2022). JNK and p38 gene and protein expression during liver ischemia-reperfusion in a rat model treated with silibinin. Iran J Basic Med Sci.

[3] Oskouei Z, Akaberi M, Hosseinzadeh H (2018). A glance at black cumin (Nigella sativa) and its active constituent, thymoquinone, in ischemia: A review. Iran J Basic Med Sci.

[4] Tania M, Asad A, Li T, Islam MS, Islam SB, Hossen MM (2021). Thymoquinone against infectious diseases: Perspectives in recent pandemics and future therapeutics. Iran J Basic Med Sci.

[5] Gulmez MI, Okuyucu S, Dokuyucu R, Gokce H (2017). The effect of caffeic acid phenethyl ester and thymoquinone on otitis media with effusion in rats. Int J Pediatr Otorhinolaryngol.

[6] Demirel H, Arli C, Ozgur T, Inci M, Dokuyucu R (2018). The role of topical thymoquinone in the treatment of acute otitis externa; an experimental study in rats. J Int Adv Otol.

[7] Verma R, Sartaj A, Qizilbash FF, Ghoneim MM, Alshehri S, Imam SS (2022). An overview of the neuropharmacological potential of thymoquinone and its targeted delivery prospects for CNS disorder. Curr Drug Metab.

[8] Islam MN, Hossain KS, Sarker PP, Ferdous J, Hannan MA, Rahman MM (2021). Revisiting pharmacological potentials of Nigella sativa seed: A promising option for COVID-19 prevention and cure. Phytother Res.

[9] Farag MM, Khalifa AA, Elhadidy WF, Rashad RM (2016). Hepatorenal protection in renal ischemia/reperfusion by celecoxib and pentoxifylline. J Surg Res.

[10] Atabay H, Demir T, Dokuyucu R, Yumrutas O, Oztuzcu S, Ceribasi A (2021). The effects of lung ischaemia/reperfusion on TRPM gene expression. West Indian Med J.

[11] Huang Q, Wang X, Lin X, Zhang J, You X, Shao A (2020). The role of transient receptor potential channels in blood-brain barrier dysfunction after ischemic stroke. Biomed Pharmacother.

[12] Cornillot M, Giacco V, Hamilton NB (2019). The role of TRP channels in white matter function and ischaemia. Neurosci Lett.

[13] Pei Z, Hu J, Bai Q, Liu B, Cheng D, Liu H (2018). Thymoquinone protects against cardiac damage from doxorubicin-induced heart failure in Sprague-Dawley rats. RSC Adv.

[14] Khbouz B, Gu S, Pinto Coelho T, Lallemand F, Jouret F (2023). Radiotherapy advances in renal disease-focus on renal ischemic preconditioning. Bioengineering (Basel).

[15] Zhang K, Xu X, Hu L (2022). Sevoflurane attenuates hepatic ischemia reperfusion injury by the miR-122/Nrf2 pathway. Ann Transl Med.

[16] Erel O, Neselioglu S (2014). A novel and automated assay for thiol/disulphide homeostasis. Clin Biochem.

[17] Erel O (2004). A novel automated direct measurement method for total antioxidant capacity using a new generation, more stable ABTS radical cation. Clin Biochem.

[18] Erel O (2005). A new automated colorimetric method for measuring total oxidant status. Clin Biochem.

[19] Cakirca G, Manav V, Celik H, Saracoglu G, Yetkin EN (2020). Effects of anxiety and depression symptoms on oxidative stress in patients with alopecia areata. Postepy Dermatol Alergol.

[20] Tolba MF, Omar HA, Azab SS, Khalifa AE, Abdel-Naim AB, Abdel-Rahman SZ (2016). Caffeic acid phenethyl ester: A review of its antioxidant activity, protective effects against ischemia-reperfusion injury and drug adverse reactions. Crit Rev Food Sci Nutr.

[21] Wu Z, Ma Y, Zhao L, Cai S, Cheng G (2018). Acute and subchronic toxicities of the ethanol and hot-water extracts from Chinese sumac (Rhus chinensis Mill ) fruits by oral administration in rats. Food Chem Toxicol.

[22] Yildiz F, Coban S, Terzi A, Ates M, Aksoy N, Cakir H (2008). Nigella sativa relieves the deleterious effects of ischemia reperfusion injury on liver. World J Gastroenterol.

[23] Huseyin S, Guclu O, Yuksel V, Erkul GSA, Can N, Turan FN (2017). Avoiding liver injury with papaverine and ascorbic acid due to infrarenal cross-clamping: An experimental study. Braz J Cardiovasc Surg.

[24] Abuzinadah MF, Ahmad A (2020). Pharmacological studies on the efficacy of a thymoquinone-containing novel polyherbal formulation against cisplatin-induced hepatorenal toxicity in rats. J Food Biochem.

[25] Abd El-Ghany RM, Sharaf NM, Kassem LA, Mahran LG, Heikal OA (2009). Thymoquinone triggers anti-apoptotic signaling targeting death ligand and apoptotic regulators in a model of hepatic ischemia reperfusion injury. Drug Discov Ther.

[26] El-Abhar HS, Abdallah DM, Saleh S (2003). Gastroprotective activity of Nigella sativa oil and its constituent, thymoquinone, against gastric mucosal injury induced by ischaemia/reperfusion in rats. J Ethnopharmacol.

[27] Bayramoglu G, Bayramoglu A, Engur S, Senturk H, Ozturk N, Colak S (2014). The hepatoprotective effects of Hypericum perforatum L on hepatic ischemia/reperfusion injury in rats. Cytotechnology.

[28] Kim SK, Jee D, Kim JY, Choi JH (2007). Effects of propofol on early phase of warm hepatic ischemia/reperfusion injury. Hepatogastroenterology.

[29] Yabe Y, Kobayashi N, Nishihashi T, Takahashi R, Nishikawa M, Takakura Y (2001). Prevention of neutrophil-mediated hepatic ischemia/reperfusion injury by superoxide dismutase and catalase derivatives. J Pharmacol Exp Ther.

[30] Serracino-Inglott F, Virlos IT, Habib NA, Williamson RC, Mathie RT (2002). Adenosine preconditioning attenuates hepatic reperfusion injury in the rat by preventing the down-regulation of endothelial nitric oxide synthase. BMC Gastroenterol.

[31] Yildirim S, Tok H, Koksal H, Erdem L, Baykan A (2002). Allopurinol plus pentoxifilline in hepatic ischaemia/reperfusion injury. Asian J Surg.

[32] Bilecik T, Karateke F, Elkan H, Gokce H (2019). The effects of TRPM2, TRPM6, TRPM7 and TRPM8 gene expression in hepatic ischemia reperfusion injury. Eur Rev Med Pharmacol Sci.

[33] Anderson KJ, Cormier RT, Scott PM (2019). Role of ion channels in gastrointestinal cancer. World J Gastroenterol.

